# Tris(2,4-dimethyl­benzene­thiol­ato)phenyl­tin(IV)

**DOI:** 10.1107/S1600536810039851

**Published:** 2010-10-13

**Authors:** Aarón Flores-Figueroa, Simón Hernández-Ortega, Ivan Castillo

**Affiliations:** a Instituto de Química, Universidad Nacional Autónoma de México, Circuito Exterior, Ciudad Universitaria, México 04510, Mexico

## Abstract

In the title compound, [Sn(C_6_H_5_)(C_8_H_9_S)_3_], the Sn atom has an approximately tetra­hedral SNCS_3_ geometry, with angles at this atom ranging from 105.13 (3) to 113.54 (9)°. The crystal packing does not involve any significant inter­molecular inter­actions, although the benzene rings are involved in a number of weak intra- and inter­molecular C—H⋯π inter­actions.

## Related literature

For background to the development of synthetic methods for highly substituted thio­phenols with varying degrees of steric hindrance, see: Lloyd-Jones *et al.* (2008 ▶); Fleischer (2005 ▶); Huber *et al.* (1997 ▶); Estudiante-Negrete *et al.* (2007 ▶). For the synthesis of phenol derivatives, see: Flores-Figueroa *et al.* (2005 ▶); Mondragón *et al.* (2010 ▶). For similar structures, see: Huber *et al.* (1997 ▶); Li *et al.* (2006 ▶). For bond-length data, see: Allen *et al.* (1987 ▶).
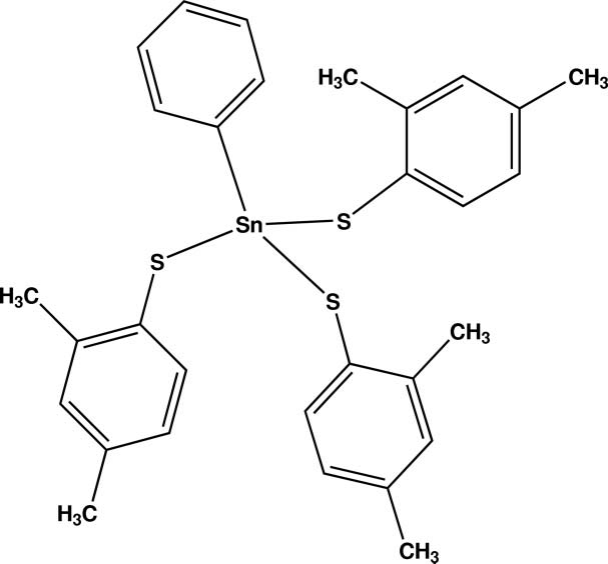


## Experimental

### 

#### Crystal data

[Sn(C_6_H_5_)(C_8_H_9_S)_3_]
*M* *_r_* = 607.43
Triclinic, 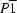

*a* = 9.2717 (7) Å
*b* = 10.6370 (8) Å
*c* = 15.6486 (11) Å
α = 93.420 (2)°
β = 93.520 (1)°
γ = 105.800 (1)°
*V* = 1477.51 (19) Å^3^
*Z* = 2
Mo *K*α radiation
μ = 1.09 mm^−1^
*T* = 298 K
0.32 × 0.26 × 0.04 mm

#### Data collection

Bruker SMART APEX CCD area-detector diffractometer
Absorption correction: multi-scan (*SADABS*; Sheldrick, 2008*a* ▶) *T*_min_ = 0.705, *T*_max_ = 0.958
12493 measured reflections
5416 independent reflections
4057 reflections with *I* > 2σ(*I*)
*R*_int_ = 0.036

#### Refinement

*R*[*F* ^2^ > 2σ(*F* ^2^)] = 0.034
*wR*(*F* ^2^) = 0.061
*S* = 0.86
5416 reflections
313 parameters
H-atom parameters constrained
Δρ_max_ = 0.64 e Å^−3^
Δρ_min_ = −0.37 e Å^−3^

### 

Data collection: *SMART* (Bruker, 2007 ▶); cell refinement: *SAINT-Plus* (Bruker, 2007 ▶); data reduction: *SAINT-Plus*; program(s) used to solve structure: *SHELXS97* (Sheldrick, 2008*b* ▶); program(s) used to refine structure: *SHELXL97* (Sheldrick, 2008*b* ▶); molecular graphics: *SHELXTL* (Sheldrick, 2008*b* ▶); software used to prepare material for publication: *SHELXTL*.

## Supplementary Material

Crystal structure: contains datablocks I, global. DOI: 10.1107/S1600536810039851/fj2342sup1.cif

Structure factors: contains datablocks I. DOI: 10.1107/S1600536810039851/fj2342Isup2.hkl

Additional supplementary materials:  crystallographic information; 3D view; checkCIF report

## Figures and Tables

**Table 1:** Hydrogen-bond geometry (Å, °) *Cg*1 and *Cg*2 are the centroids of the C15–C20 and C7–C12 rings, respectively.

*D*—H⋯*A*	*D*—H	H⋯*A*	*D*⋯*A*	*D*—H⋯*A*
C12—H12⋯*Cg*1	0.93	2.72	3.557 (3)	149
C13—H13*C*⋯*Cg*2^i^	0.96	2.75	3.579 (3)	144

Symmetry code: (i) 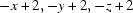.

**Fig. 1.**
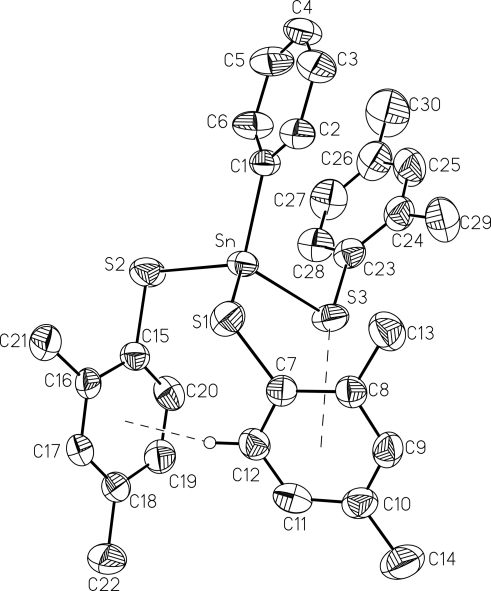
The molecular structure of PhSn(SMe2Ph)3 with numbering scheme. Displacement ellipsoids are drawn at the 40% probability level. Only the H atoms involved in C—H-π and S-π interactions are shown.

## supplementary crystallographic information

### Comment

The development of synthetic methods for highly substituted thiophenols with varying degrees of steric hindrance has been an active field of research (Lloyd-Jones *et al.*, 2008), due in part to the potential of sterically encumbered thiophenols to emulate the active site of sulfur-rich metalloenzymes (Fleischer, 2005). In this context, we developed a series of 2,4-disubstituted thiophenols (Flores-Figueroa *et al.*, 2005, Mondragón *et al.*,2010), among which 2,4-dimethylthiophenol represents a commercially available ligand. In order to assess the steric properties of this thiophenol, we soughtout to prepare a metal-thiolate derivative amenable to structural characterization. Since phenyl- and diphenyltin (IV) derivatives tend to be crystalline materials (Huber *et al.*, 1997 & Estudiante-Negrete *et al.*, 2007), we decided to employ PhSnCl_3_ to introduce the 2,4-dimethylthiophenolate moiety. Thus, the reaction of 3 equivalents of 2,4-Me_2_C_6_H_3_SH with PhSnCl_3_ in the presence of 3 equivalents of triethylamine afforded the title compound phenyl tris(2,4-dimethylphenylthiolate)tin (IV) (I) in good yield.

The structure of the title compound (PhSn(S-2,4-Me_2_C_6_H_3_)_3_,) is shown with numbering scheme in Figure 1. According to the bond angles, (I) exhibits a slightly distorted tetrahedral geometry. The phenyl ring (C1—C6) is in a close to coplanar disposition with respect to one of the 2,4-dimethylphenyl groups (C23–C30), forming a dihedral angle of 25.3 (2)°. The Sn—C distance (2.114 (3) Å) is slightly shorter than those described for the related compounds (Allen *et al.*, 1987) phenyl tris(pyridinthiolate)tin [2.139 (5) Å, PhSn(SPy)_3_ (Huber *et al.*, 1997)] and phenyl tris(pyrimidinethiolato)tin [2.139 (3) Å, PhSn(SPym)_3_ (Li *et al.*, 2006)]. The Sn—S distances are shorter than those in PhSn(SPy)_3_ [2.491–2.576 Å], and PhSn(SPym)_3_[2.455–2.552 Å]. Due to the geometry adopted, in the crystal structure, there are C—H-π, intra and intermolecular interactions.

### Experimental

To a tetrahydrofuran solution of 2,4-dimethylthiophenol (0.50 g, 3.70 mmol) was added triethylamine (0.65 ml, 4.07 mmol) under an atmosphere of N_2_. After stirring for 1 h, PhSnCl_3_ (0.20 mL, 1.23 mmol) was added *via* syringe, and the mixture was stirred overnight. The volatile materials were evaporated under reduced pressure, and the solid was extracted with hexane (2 *x* 15 ml), and X-ray quality crystals were obtained by slow evaporation of the solution. Yield: 0.48 g (64%); m.p. 320–323 K; IR (KBr, cm^-1^) 3056, 3012, 2916, 2859, 2728, 1898, 1753, 1598, 1471, 1434, 1373, 1266,1229, 1162, 1138, 1069, 1042, 876, 811, 728, 695, 620, 543, 443, 372, 299; ^1^H NMR (300 MHz, CDCl_3_, TMS internal reference δ p.p.m.)7.20 (2*H*, m, Ph) 7.11 (3*H*, d,ArH), 7.08 (1*H*, s, Ph), 6.95 (2*H*, d, Ph), 6.85 (3*H*, s, ArH), 6.69 (3*H*, d,ArH), 2.18 (18*H*, s, ArMe); ^13^C NMR (75 MHz, CDCl_3_, TMS internal reference, δ p.p.m.)142.29, 139.48, 137.62, 136. 60, 135.17, 131.58, 130.44, 128.69, 127.32,123.88, 22.19, 21.03.

### Refinement

H atoms were included in calculated positions (C—H = 0.93Å arom, and 0.96 Å CH_3_), and refined using a riding model, with *U*ĩso~(H) = 1.2*U*_eq_ and 1.5 *U*_eq_ respectively of the carrier atom.

### Crystal data

**Table:**

[Sn(C_6_H_5_)(C_8_H_9_S)_3_]	*Z* = 2
*M**_r_* = 607.43	*F*(000) = 620
Triclinic, *P*1	*D*_x_ = 1.365 Mg m^−^^3^
Hall symbol: -P 1	Mo *K*α radiation, λ = 0.71073 Å
*a* = 9.2717 (7) Å	Cell parameters from 6072 reflections
*b* = 10.6370 (8) Å	θ = 2.3–25.4°
*c* = 15.6486 (11) Å	µ = 1.09 mm^−^^1^
α = 93.420 (2)°	*T* = 298 K
β = 93.520 (1)°	Prism-lamina, colourless
γ = 105.800 (1)°	0.32 × 0.26 × 0.04 mm
*V* = 1477.51 (19) Å^3^	

### Data collection

**Table:**

Bruker SMART APEX CCD area-detector diffractometer	5416 independent reflections
Radiation source: fine-focus sealed tube	4057 reflections with *I* > 2σ(*I*)
graphite	*R*_int_ = 0.036
Detector resolution: 0.83 pixels mm^-1^	θ_max_ = 25.4°, θ_min_ = 1.3°
ω scans	*h* = −11→11
Absorption correction: multi-scan (*SADABS*; Sheldrick, 2008*a*)	*k* = −12→12
*T*_min_ = 0.705, *T*_max_ = 0.958	*l* = −18→18
12493 measured reflections	

### Refinement

**Table:**

Refinement on *F*^2^	Primary atom site location: structure-invariant direct methods
Least-squares matrix: full	Secondary atom site location: difference Fourier map
*R*[*F*^2^ > 2σ(*F*^2^)] = 0.034	Hydrogen site location: inferred from neighbouring sites
*wR*(*F*^2^) = 0.061	H-atom parameters constrained
*S* = 0.86	*w* = 1/[σ^2^(*F*_o_^2^) + (0.021*P*)^2^] where *P* = (*F*_o_^2^ + 2*F*_c_^2^)/3
5416 reflections	(Δ/σ)_max_ = 0.002
313 parameters	Δρ_max_ = 0.64 e Å^−^^3^
0 restraints	Δρ_min_ = −0.37 e Å^−^^3^

### Special details

**Table:**

Geometry. All e.s.d.'s (except the e.s.d. in the dihedral angle between two l.s. planes) are estimated using the full covariance matrix. The cell e.s.d.'s are taken into account individually in the estimation of e.s.d.'s in distances, angles and torsion angles; correlations between e.s.d.'s in cell parameters are only used when they are defined by crystal symmetry. An approximate (isotropic) treatment of cell e.s.d.'s is used for estimating e.s.d.'s involving l.s. planes.
Refinement. Refinement of *F*^2^ against ALL reflections. The weighted *R*-factor *wR* and goodness of fit *S* are based on *F*^2^, conventional *R*-factors *R* are based on *F*, with *F* set to zero for negative *F*^2^. The threshold expression of *F*^2^ > σ(*F*^2^) is used only for calculating *R*-factors(gt) *etc*. and is not relevant to the choice of reflections for refinement. *R*-factors based on *F*^2^ are statistically about twice as large as those based on *F*, and *R*- factors based on ALL data will be even larger.

### Fractional atomic coordinates and isotropic or equivalent isotropic displacement parameters (Å^2^)

**Table:**

	*x*	*y*	*z*	*U*_iso_*/*U*_eq_	
Sn	0.76585 (2)	0.68776 (2)	0.732737 (14)	0.04856 (8)	
S1	0.79793 (10)	0.74342 (8)	0.88495 (5)	0.0604 (2)	
S2	0.76443 (10)	0.46303 (9)	0.70323 (6)	0.0713 (3)	
S3	0.97179 (9)	0.83866 (9)	0.67586 (6)	0.0649 (3)	
C1	0.5620 (3)	0.7073 (3)	0.6745 (2)	0.0493 (8)	
C2	0.4930 (4)	0.7944 (3)	0.7097 (2)	0.0613 (9)	
H2	0.5331	0.8414	0.7617	0.074*	
C3	0.3652 (4)	0.8137 (4)	0.6695 (3)	0.0784 (11)	
H3	0.3188	0.8721	0.6949	0.094*	
C4	0.3072 (4)	0.7475 (4)	0.5931 (3)	0.0784 (12)	
H4	0.2211	0.7607	0.5659	0.094*	
C5	0.3745 (4)	0.6615 (4)	0.5557 (2)	0.0799 (12)	
H5	0.3355	0.6172	0.5027	0.096*	
C6	0.5015 (4)	0.6405 (4)	0.5974 (2)	0.0698 (10)	
H6	0.5460	0.5803	0.5726	0.084*	
C7	0.9858 (3)	0.8485 (3)	0.88924 (18)	0.0480 (8)	
C8	1.0125 (4)	0.9837 (3)	0.88760 (18)	0.0503 (8)	
C9	1.1612 (4)	1.0570 (3)	0.89292 (19)	0.0615 (9)	
H9	1.1811	1.1475	0.8925	0.074*	
C10	1.2818 (4)	1.0050 (4)	0.8988 (2)	0.0627 (9)	
C11	1.2497 (4)	0.8703 (4)	0.8994 (2)	0.0652 (10)	
H11	1.3281	0.8313	0.9029	0.078*	
C12	1.1045 (4)	0.7937 (3)	0.89512 (19)	0.0558 (9)	
H12	1.0855	0.7034	0.8961	0.067*	
C13	0.8877 (4)	1.0482 (3)	0.8784 (2)	0.0729 (10)	
H13A	0.8203	1.0071	0.8294	0.109*	
H13B	0.9292	1.1395	0.8707	0.109*	
H13C	0.8338	1.0392	0.9292	0.109*	
C14	1.4406 (4)	1.0917 (4)	0.9042 (3)	0.1016 (14)	
H14A	1.4584	1.1336	0.8517	0.152*	
H14B	1.5094	1.0398	0.9131	0.152*	
H14C	1.4553	1.1572	0.9513	0.152*	
C15	0.9354 (3)	0.4663 (3)	0.7653 (2)	0.0525 (8)	
C16	0.9311 (3)	0.4193 (3)	0.8463 (2)	0.0519 (8)	
C17	1.0672 (4)	0.4228 (3)	0.8900 (2)	0.0564 (9)	
H17	1.0661	0.3920	0.9444	0.068*	
C18	1.2041 (4)	0.4697 (3)	0.8564 (2)	0.0584 (9)	
C19	1.2029 (4)	0.5147 (3)	0.7760 (2)	0.0673 (10)	
H19	1.2935	0.5469	0.7517	0.081*	
C20	1.0702 (4)	0.5132 (3)	0.7307 (2)	0.0645 (9)	
H20	1.0720	0.5441	0.6763	0.077*	
C21	0.7866 (4)	0.3664 (3)	0.8865 (2)	0.0773 (11)	
H21A	0.7355	0.4334	0.8922	0.116*	
H21B	0.8074	0.3391	0.9422	0.116*	
H21C	0.7240	0.2928	0.8508	0.116*	
C22	1.3519 (4)	0.4742 (4)	0.9061 (2)	0.0858 (12)	
H22A	1.4186	0.4506	0.8676	0.129*	
H22B	1.3332	0.4137	0.9500	0.129*	
H22C	1.3970	0.5613	0.9322	0.129*	
C23	0.8789 (3)	0.8297 (3)	0.5717 (2)	0.0579 (9)	
C24	0.8116 (4)	0.9252 (3)	0.5474 (2)	0.0661 (10)	
C25	0.7413 (5)	0.9110 (4)	0.4651 (3)	0.0882 (13)	
H25	0.6951	0.9741	0.4485	0.106*	
C26	0.7372 (5)	0.8077 (5)	0.4068 (3)	0.0920 (14)	
C27	0.8052 (5)	0.7156 (4)	0.4322 (3)	0.0902 (13)	
H27	0.8037	0.6447	0.3941	0.108*	
C28	0.8759 (4)	0.7266 (4)	0.5136 (2)	0.0759 (11)	
H28	0.9223	0.6632	0.5294	0.091*	
C29	0.8112 (5)	1.0413 (4)	0.6079 (3)	0.0950 (13)	
H29A	0.7519	1.0114	0.6547	0.143*	
H29B	0.7690	1.1005	0.5776	0.143*	
H29C	0.9124	1.0857	0.6299	0.143*	
C30	0.6584 (6)	0.7974 (5)	0.3179 (3)	0.146 (2)	
H30A	0.5860	0.8471	0.3188	0.219*	
H30B	0.6081	0.7072	0.3006	0.219*	
H30C	0.7310	0.8315	0.2780	0.219*	

### Atomic displacement parameters (Å^2^)

**Table:**

	*U*^11^	*U*^22^	*U*^33^	*U*^12^	*U*^13^	*U*^23^
Sn	0.04127 (13)	0.05660 (15)	0.04794 (14)	0.01525 (11)	−0.00392 (9)	0.00492 (10)
S1	0.0608 (6)	0.0633 (6)	0.0494 (5)	0.0041 (5)	0.0036 (4)	0.0058 (4)
S2	0.0706 (6)	0.0607 (6)	0.0782 (7)	0.0212 (5)	−0.0255 (5)	−0.0114 (5)
S3	0.0442 (5)	0.0874 (7)	0.0578 (6)	0.0085 (5)	0.0002 (4)	0.0123 (5)
C1	0.0378 (18)	0.057 (2)	0.053 (2)	0.0136 (16)	0.0015 (15)	0.0104 (17)
C2	0.050 (2)	0.066 (2)	0.069 (2)	0.0185 (19)	−0.0010 (18)	0.0057 (19)
C3	0.060 (3)	0.080 (3)	0.104 (3)	0.036 (2)	0.005 (2)	0.009 (2)
C4	0.048 (2)	0.098 (3)	0.094 (3)	0.026 (2)	−0.006 (2)	0.034 (3)
C5	0.058 (2)	0.112 (3)	0.063 (3)	0.021 (2)	−0.0156 (19)	−0.003 (2)
C6	0.050 (2)	0.089 (3)	0.074 (3)	0.029 (2)	−0.0029 (19)	−0.008 (2)
C7	0.057 (2)	0.047 (2)	0.0360 (18)	0.0095 (17)	−0.0030 (15)	−0.0001 (14)
C8	0.062 (2)	0.049 (2)	0.0385 (18)	0.0144 (18)	−0.0052 (15)	0.0018 (15)
C9	0.081 (3)	0.046 (2)	0.048 (2)	0.005 (2)	−0.0101 (18)	0.0026 (16)
C10	0.057 (2)	0.073 (3)	0.050 (2)	0.005 (2)	−0.0043 (17)	0.0099 (19)
C11	0.062 (2)	0.083 (3)	0.054 (2)	0.028 (2)	−0.0076 (17)	0.0047 (19)
C12	0.066 (2)	0.052 (2)	0.049 (2)	0.017 (2)	−0.0063 (17)	0.0040 (16)
C13	0.087 (3)	0.061 (2)	0.074 (3)	0.031 (2)	−0.008 (2)	0.0000 (19)
C14	0.069 (3)	0.115 (3)	0.098 (3)	−0.014 (3)	−0.015 (2)	0.027 (3)
C15	0.054 (2)	0.0442 (19)	0.060 (2)	0.0171 (17)	−0.0060 (17)	−0.0017 (16)
C16	0.051 (2)	0.0379 (18)	0.067 (2)	0.0131 (16)	0.0047 (17)	0.0106 (16)
C17	0.059 (2)	0.047 (2)	0.065 (2)	0.0169 (18)	0.0013 (18)	0.0151 (17)
C18	0.053 (2)	0.047 (2)	0.077 (3)	0.0186 (18)	−0.0001 (19)	0.0082 (18)
C19	0.052 (2)	0.066 (2)	0.090 (3)	0.0209 (19)	0.020 (2)	0.018 (2)
C20	0.074 (3)	0.069 (2)	0.060 (2)	0.030 (2)	0.014 (2)	0.0170 (18)
C21	0.061 (2)	0.068 (2)	0.103 (3)	0.013 (2)	0.011 (2)	0.029 (2)
C22	0.059 (2)	0.087 (3)	0.113 (3)	0.027 (2)	−0.013 (2)	0.010 (2)
C23	0.052 (2)	0.068 (2)	0.050 (2)	0.0091 (19)	0.0078 (16)	0.0087 (19)
C24	0.075 (3)	0.062 (2)	0.054 (2)	0.005 (2)	0.0029 (19)	0.0147 (19)
C25	0.106 (3)	0.079 (3)	0.075 (3)	0.018 (3)	−0.007 (3)	0.027 (2)
C26	0.113 (4)	0.091 (3)	0.055 (3)	0.002 (3)	−0.007 (2)	0.015 (3)
C27	0.116 (4)	0.089 (3)	0.058 (3)	0.020 (3)	0.007 (2)	−0.008 (2)
C28	0.074 (3)	0.090 (3)	0.066 (3)	0.025 (2)	0.009 (2)	0.006 (2)
C29	0.125 (4)	0.072 (3)	0.092 (3)	0.031 (3)	0.005 (3)	0.015 (2)
C30	0.197 (6)	0.145 (5)	0.072 (3)	0.017 (4)	−0.044 (3)	0.016 (3)

### Geometric parameters (Å, °)

**Table:**

Sn—C1	2.114 (3)	C15—C20	1.371 (4)
Sn—S3	2.3927 (9)	C15—C16	1.390 (4)
Sn—S1	2.4002 (9)	C16—C17	1.388 (4)
Sn—S2	2.4037 (9)	C16—C21	1.498 (4)
S1—C7	1.790 (3)	C17—C18	1.380 (4)
S2—C15	1.798 (3)	C17—H17	0.9300
S3—C23	1.782 (3)	C18—C19	1.373 (4)
C1—C6	1.368 (4)	C18—C22	1.521 (4)
C1—C2	1.370 (4)	C19—C20	1.378 (4)
C2—C3	1.378 (4)	C19—H19	0.9300
C2—H2	0.9300	C20—H20	0.9300
C3—C4	1.353 (5)	C21—H21A	0.9600
C3—H3	0.9300	C21—H21B	0.9600
C4—C5	1.363 (5)	C21—H21C	0.9600
C4—H4	0.9300	C22—H22A	0.9600
C5—C6	1.389 (4)	C22—H22B	0.9600
C5—H5	0.9300	C22—H22C	0.9600
C6—H6	0.9300	C23—C28	1.375 (4)
C7—C12	1.379 (4)	C23—C24	1.388 (4)
C7—C8	1.394 (4)	C24—C25	1.388 (5)
C8—C9	1.381 (4)	C24—C29	1.512 (5)
C8—C13	1.501 (4)	C25—C26	1.376 (5)
C9—C10	1.377 (4)	C25—H25	0.9300
C9—H9	0.9300	C26—C27	1.365 (5)
C10—C11	1.382 (4)	C26—C30	1.516 (5)
C10—C14	1.504 (4)	C27—C28	1.380 (5)
C11—C12	1.365 (4)	C27—H27	0.9300
C11—H11	0.9300	C28—H28	0.9300
C12—H12	0.9300	C29—H29A	0.9600
C13—H13A	0.9600	C29—H29B	0.9600
C13—H13B	0.9600	C29—H29C	0.9600
C13—H13C	0.9600	C30—H30A	0.9600
C14—H14A	0.9600	C30—H30B	0.9600
C14—H14B	0.9600	C30—H30C	0.9600
C14—H14C	0.9600		
			
C1—Sn—S3	108.97 (8)	C16—C15—S2	120.6 (3)
C1—Sn—S1	113.54 (9)	C17—C16—C15	117.5 (3)
S3—Sn—S1	105.13 (3)	C17—C16—C21	120.3 (3)
C1—Sn—S2	106.68 (9)	C15—C16—C21	122.2 (3)
S3—Sn—S2	112.83 (4)	C18—C17—C16	123.1 (3)
S1—Sn—S2	109.83 (3)	C18—C17—H17	118.5
C7—S1—Sn	97.37 (10)	C16—C17—H17	118.5
C15—S2—Sn	98.83 (10)	C19—C18—C17	117.4 (3)
C23—S3—Sn	95.05 (11)	C19—C18—C22	120.6 (3)
C6—C1—C2	118.1 (3)	C17—C18—C22	122.0 (3)
C6—C1—Sn	120.8 (2)	C18—C19—C20	121.3 (3)
C2—C1—Sn	120.9 (2)	C18—C19—H19	119.4
C1—C2—C3	121.3 (3)	C20—C19—H19	119.4
C1—C2—H2	119.3	C15—C20—C19	120.4 (3)
C3—C2—H2	119.3	C15—C20—H20	119.8
C4—C3—C2	119.8 (4)	C19—C20—H20	119.8
C4—C3—H3	120.1	C16—C21—H21A	109.5
C2—C3—H3	120.1	C16—C21—H21B	109.5
C3—C4—C5	120.3 (3)	H21A—C21—H21B	109.5
C3—C4—H4	119.8	C16—C21—H21C	109.5
C5—C4—H4	119.8	H21A—C21—H21C	109.5
C4—C5—C6	119.6 (4)	H21B—C21—H21C	109.5
C4—C5—H5	120.2	C18—C22—H22A	109.5
C6—C5—H5	120.2	C18—C22—H22B	109.5
C1—C6—C5	120.8 (3)	H22A—C22—H22B	109.5
C1—C6—H6	119.6	C18—C22—H22C	109.5
C5—C6—H6	119.6	H22A—C22—H22C	109.5
C12—C7—C8	120.2 (3)	H22B—C22—H22C	109.5
C12—C7—S1	119.0 (2)	C28—C23—C24	119.4 (3)
C8—C7—S1	120.8 (3)	C28—C23—S3	119.0 (3)
C9—C8—C7	116.6 (3)	C24—C23—S3	121.6 (3)
C9—C8—C13	120.8 (3)	C23—C24—C25	118.1 (4)
C7—C8—C13	122.5 (3)	C23—C24—C29	122.1 (3)
C10—C9—C8	124.3 (3)	C25—C24—C29	119.8 (4)
C10—C9—H9	117.8	C26—C25—C24	122.8 (4)
C8—C9—H9	117.8	C26—C25—H25	118.6
C9—C10—C11	116.9 (3)	C24—C25—H25	118.6
C9—C10—C14	121.0 (4)	C27—C26—C25	117.9 (4)
C11—C10—C14	122.0 (4)	C27—C26—C30	121.8 (5)
C12—C11—C10	120.9 (3)	C25—C26—C30	120.4 (5)
C12—C11—H11	119.5	C26—C27—C28	120.9 (4)
C10—C11—H11	119.5	C26—C27—H27	119.6
C11—C12—C7	121.0 (3)	C28—C27—H27	119.6
C11—C12—H12	119.5	C23—C28—C27	121.0 (4)
C7—C12—H12	119.5	C23—C28—H28	119.5
C8—C13—H13A	109.5	C27—C28—H28	119.5
C8—C13—H13B	109.5	C24—C29—H29A	109.5
H13A—C13—H13B	109.5	C24—C29—H29B	109.5
C8—C13—H13C	109.5	H29A—C29—H29B	109.5
H13A—C13—H13C	109.5	C24—C29—H29C	109.5
H13B—C13—H13C	109.5	H29A—C29—H29C	109.5
C10—C14—H14A	109.5	H29B—C29—H29C	109.5
C10—C14—H14B	109.5	C26—C30—H30A	109.5
H14A—C14—H14B	109.5	C26—C30—H30B	109.5
C10—C14—H14C	109.5	H30A—C30—H30B	109.5
H14A—C14—H14C	109.5	C26—C30—H30C	109.5
H14B—C14—H14C	109.5	H30A—C30—H30C	109.5
C20—C15—C16	120.3 (3)	H30B—C30—H30C	109.5
C20—C15—S2	119.0 (3)		
			
C1—Sn—S1—C7	−131.63 (14)	C14—C10—C11—C12	179.5 (3)
S3—Sn—S1—C7	−12.61 (11)	C10—C11—C12—C7	0.6 (5)
S2—Sn—S1—C7	109.04 (11)	C8—C7—C12—C11	0.0 (5)
C1—Sn—S2—C15	−175.21 (14)	S1—C7—C12—C11	−179.3 (2)
S3—Sn—S2—C15	65.16 (12)	Sn—S2—C15—C20	−81.6 (3)
S1—Sn—S2—C15	−51.77 (12)	Sn—S2—C15—C16	99.9 (2)
C1—Sn—S3—C23	−32.90 (16)	C20—C15—C16—C17	0.4 (5)
S1—Sn—S3—C23	−154.93 (13)	S2—C15—C16—C17	178.9 (2)
S2—Sn—S3—C23	85.39 (13)	C20—C15—C16—C21	−179.6 (3)
S3—Sn—C1—C6	85.5 (3)	S2—C15—C16—C21	−1.1 (4)
S1—Sn—C1—C6	−157.8 (2)	C15—C16—C17—C18	−0.3 (5)
S2—Sn—C1—C6	−36.6 (3)	C21—C16—C17—C18	179.7 (3)
S3—Sn—C1—C2	−89.7 (3)	C16—C17—C18—C19	0.1 (5)
S1—Sn—C1—C2	27.1 (3)	C16—C17—C18—C22	179.1 (3)
S2—Sn—C1—C2	148.2 (2)	C17—C18—C19—C20	0.0 (5)
C6—C1—C2—C3	0.7 (5)	C22—C18—C19—C20	−179.0 (3)
Sn—C1—C2—C3	176.0 (3)	C16—C15—C20—C19	−0.3 (5)
C1—C2—C3—C4	−1.1 (6)	S2—C15—C20—C19	−178.8 (3)
C2—C3—C4—C5	0.2 (6)	C18—C19—C20—C15	0.1 (5)
C3—C4—C5—C6	1.1 (6)	Sn—S3—C23—C28	−81.4 (3)
C2—C1—C6—C5	0.6 (5)	Sn—S3—C23—C24	99.6 (3)
Sn—C1—C6—C5	−174.6 (3)	C28—C23—C24—C25	1.0 (5)
C4—C5—C6—C1	−1.6 (6)	S3—C23—C24—C25	−179.9 (3)
Sn—S1—C7—C12	−85.7 (2)	C28—C23—C24—C29	−179.8 (3)
Sn—S1—C7—C8	95.0 (2)	S3—C23—C24—C29	−0.8 (5)
C12—C7—C8—C9	−0.6 (4)	C23—C24—C25—C26	−0.7 (6)
S1—C7—C8—C9	178.7 (2)	C29—C24—C25—C26	−179.8 (4)
C12—C7—C8—C13	178.0 (3)	C24—C25—C26—C27	0.3 (7)
S1—C7—C8—C13	−2.7 (4)	C24—C25—C26—C30	179.9 (4)
C7—C8—C9—C10	0.6 (5)	C25—C26—C27—C28	−0.2 (7)
C13—C8—C9—C10	−178.0 (3)	C30—C26—C27—C28	−179.8 (4)
C8—C9—C10—C11	−0.1 (5)	C24—C23—C28—C27	−1.0 (5)
C8—C9—C10—C14	179.9 (3)	S3—C23—C28—C27	179.9 (3)
C9—C10—C11—C12	−0.6 (5)	C26—C27—C28—C23	0.6 (6)

### Hydrogen-bond geometry (Å, °)

**Table:**

Cg1 and Cg2 are the centroids of the C15–C20 and C7–C12 rings, respectively.

**Table:**

*D*—H···*A*	*D*—H	H···*A*	*D*···*A*	*D*—H···*A*
C12—H12···Cg1	0.93	2.72	3.557 (3)	149
C13—H13C···Cg2^i^	0.96	2.75	3.579 (3)	144

Symmetry codes: (i) −*x*+2, −*y*+2, −*z*+2.

## References

[bb1] Allen, F. H. (2002). *Acta Cryst.* B**58**, 380–388. 10.1107/s0108768102003890 12037359

[bb2] Allen, F. H., Kennard, O., Watson, D. G., Brammer, L., Orpen, A. G. & Taylor, R. (1987). *J. Chem. Soc. Perkin Trans. 2*, pp. S1–19.

[bb3] Bruker (2007). *SMART* and *SAINT-Plus* Bruker AXS Inc., Madison, Wisconsin, USA.

[bb4] Estudiante-Negrete, F., Redón, R., Hernández-Ortega, S., Toscano, R. A. & Morales-Morales, D. (2007). *Inorg. Chim. Acta*, **360**, 1651–1660.

[bb5] Fleischer, H. (2005). *Coord. Chem. Rev.* **249**, 799–827.

[bb6] Flores-Figueroa, A., Arista-M, V., Talancón-Sánchez, D. & Castillo, I. (2005). *J. Braz. Chem. Soc* **16**, 397–403.

[bb7] Huber, F., Schmiedgen, R., Schurmann, M., Barbieri, R., Ruisi, G. & Silvestri, A. (1997). *Appl. Organomet. Chem.* **11**, 869–888.

[bb8] Li, Y.-X., Zhang, R.-F. & Ma, C.-L. (2006). *Acta Cryst.* E**62**, m957–m958.

[bb9] Lloyd-Jones, G. C., Moseley, J. D. & Renny, J. S. (2008). *Synthesis*, pp. 661–689.

[bb10] Mondragón, A., Monsalvo, I., Regla, I. & Castillo, I. (2010). *Tetrahedron Lett.* **51**, 767–770.

[bb11] Sheldrick, G. M. (2008*a*). *SADABS* University of Göttingen, Germany.

[bb12] Sheldrick, G. M. (2008*b*). *Acta Cryst.* A**64**, 112–122. 10.1107/S0108767307043930 18156677

